# Screening for modulators of neural network activity in 3D human iPSC-derived cortical spheroids

**DOI:** 10.1371/journal.pone.0240991

**Published:** 2020-10-22

**Authors:** Grace Woodruff, Naomi Phillips, Cassiano Carromeu, Oivin Guicherit, Alistair White, McCay Johnson, Fabian Zanella, Blake Anson, Timothy Lovenberg, Pascal Bonaventure, Anthony W. Harrington

**Affiliations:** 1 Neuroscience Discovery, Janssen Research and Development, LLC., San Diego, California, United States of America; 2 StemoniX, Inc, Maple Grove, Minnesota, United States of America; New York Medical College, UNITED STATES

## Abstract

Human induced Pluripotent Stem Cells (iPSCs) are a powerful tool to dissect the biology of complex human cell types such as those of the central nervous system (CNS). However, robust, high-throughput platforms for reliably measuring activity in human iPSC-derived neuronal cultures are lacking. Here, we assessed 3D cultures of cortical neurons and astrocytes displaying spontaneous, rhythmic, and highly synchronized neural activity that can be visualized as calcium oscillations on standard high-throughput fluorescent readers as a platform for CNS-based discovery efforts. Spontaneous activity and spheroid structure were highly consistent from well-to-well, reference compounds such as TTX, 4-AP, AP5, and NBQX, had expected effects on neural spontaneous activity, demonstrating the presence of functionally integrated neuronal circuitry. Neurospheroid biology was challenged by screening the LOPAC^®1280^ library, a collection of 1280 pharmacologically active small molecules. The primary screen identified 111 compounds (8.7%) that modulated neural network activity across a wide range of neural and cellular processes and 16 of 17 compounds chosen for follow-up confirmed the primary screen results. Together, these data demonstrate the suitability and utility of human iPSC-derived neurospheroids as a screening platform for CNS-based drug discovery.

## Introduction

Modern conventional high-throughput drug screening typically uses recombinant cell lines that overexpress a drug target of interest. The advent of human induced pluripotent stem cells (iPSCs) promises, among other things, the development of relevant cellular disease models for use in high-throughput screening. Human iPSCs offer many advantages over recombinant cell lines or primary rodent cells for use in drug screening. Because these cells are derived from human donors, human genetic diseases can be more accurately modeled, especially when used in combination with modern genome editing techniques. Furthermore, the ability of iPSCs to be differentiated into a variety of cell types further expands their utility, as screens can be conducted using functional or disease-relevant assays in an appropriate cell type that recapitulates many of the native cellular processes. This type of screening has the potential to reveal novel biology and lead to new pharmacological mechanisms of action. Disease modeling in iPSCs has improved our understanding of critical pathways for a variety of neurological diseases including Rett syndrome, Alzheimer’s Disease (AD), and schizophrenia [1–6]. Many psychiatric diseases are hypothesized to be caused by defects in the normal circuitry and excitatory properties of neurons in the brain, while AD is characterized by synaptic loss and dysfunction [7–11]. Consequently, development of a human iPSC-based high throughput screening platform to identify compounds that modulate neuronal activity and network connectivity is of potential therapeutic value.

Cultures of rat primary neurons *in vitro* form synaptic networks that generate synchronized oscillations in the levels of cytoplasmic calcium [12]. These oscillations correspond to bursts of network activity and can be modulated with excitatory and inhibitory compounds [13, 14]. As network activity results from several inter-connected processes, including structural interactions, cell health, neuronal firing, synaptic transmission, and a variety of additional cellular processes; Ca^2+^ oscillations can act as a global phenotypic readout for normal and pathophysiological functions. *In vitro*, these oscillations can be detected by measurement of calcium concentration fluctuations using kinetic fluorometric imaging plate reader instruments (such as the FLIPR Tetra^®^ from Molecular Devices or the FDSS^®^/μCell from Hamamatsu), thus making Ca^2+^ oscillations amenable for high-throughput screening [15]. However, the use of rodent neurons for drug screening may not translate to human neurons, and therefore a method for measuring activity in human neurons is desired. Platforms for measuring spontaneous activity in human iPSC-derived neurons, such as multi electrode array (MEA), provide detailed measurements of individual neurons and network activity. However, MEA measurements can be variable in human iPSC neuronal cultures and are currently restricted to 96-wells, which limits the utility of MEA in high-throughput screening.

In this study, 3D human iPSC-derived cultures were assessed as suitable biological substrates to screen for modulators of spontaneous neuronal network activity. Human iPSC-derived cultures of neurons and astrocytes generate measurable spontaneous calcium oscillations in both two-dimensional (2D) cultures and three-dimensional (3D) spheroids. Well-to-well oscillatory activity was much more reproducible in 3D spheroids, in part due to the nature of 2D activity being below the level of detection, thus making the 3D platform better suited for high-throughput screening. As expected, network oscillations were modulated by multiple neuroactive compounds, demonstrating functional neuronal networks. The Library of Pharmacologically Active Compounds (LOPAC^®1280^), which contains 1280 well-characterized compounds with annotated activity, was then used to test the utility of this platform for drug discovery where Ca^2+^ oscillation frequency and amplitude were quantified at 30 minutes, 2 hours, and 4 hours following compound treatment. From the library, 111 (8.7%) compounds were identified that altered frequency or amplitude in at least one timepoint. Of the 111 hits, 17 (~15%) of them altered calcium oscillation frequency and amplitude at all timepoints and these were tested for concentration response, where 16 of the 17 selected compounds confirmed the primary screen results. Our results demonstrate the usefulness of this assay in high-throughput screening and provides an innovative approach for CNS-based drug discovery in a relevant human 3D system.

## Materials and methods

### Human iPSC-derived 3D neural cultures

Assay-ready 384-well microBrain 3D cultures of human neurons and astrocytes were obtained from StemoniX, Inc. (BSARX-AA-0384). The source iPSCs were from a male individual without any known deleterious mutations and a normal healthy phenotype. As described previously [16], the microBrain 3D platform was generated, differentiated, and matured for 8 weeks prior to shipment. Over the time of differentiation and maturity, the spheroids spontaneously develop synchronized calcium activity. Upon receipt, the 3D co-differentiated population of cortical neurons and astrocytes [16] was maintained in and replenished every two days for one week prior to assay with BrainPhys (StemCell Technologies, 05790) media supplemented with 1x SM1 (StemCell Technologies, 05711), 20ng/mL of BDNF (StemCell Technologies, 78005), 20ng/mL of GDNF (StemCell Technologies, 78058), and 1x pen/strep (Corning, 30-002-CI).

### Immunofluorescence

3D neurospheres were fixed in 4% paraformaldehyde solution in PBS for 10 minutes at room temperature. Next, neurospheres were washed 5x with half volume changes of PBS. Neurospheres were permeabilized in 0.4% Triton-X in Odyssey Blocking Buffer (LiCor) for 15 minutes at room temperature followed by addition of blocking solution (Odyssey Blocking Buffer + 0.1% Triton-X) and incubated for 4 hours at room temperature. After blocking, primary antibodies (MAP2 from Millipore, MAB3418; GFAP from Abcam, ab134436) were added at 1:250 dilution in blocking solution and incubated overnight at 4°C with gentle agitation. The next day, neurospheres were washed 8x with half washes of PBS + 0.1% Tween-20 with 2 minutes for each wash. Secondary antibodies were then added in blocking solution and incubated for 1 hour at room temperature, protected from light. After the secondary antibody incubation, neurospheres were washed 4x with half washes of PBS + 0.1% Tween-20. The nuclei were stained using DAPI for 8 minutes at room temperature followed by 8 half washes with PBS. Neurospheres were imaged in PBS using an ImageXpress Confocal Microscope (Molecular Devices) at 20x magnification.

### Lentiviral transduction and neuronal calcium activity recording

Neurospheroids were transduced with Incucyte Neuroburst Orange reagent (Satorius, 4736). Neuroburst Orange reagent is a lentivirus genetically encoding a fluorescence calcium indicator driven by the synapsin promoter. For the labeling, Neuroburst Orange reagent was added to individual wells of the microBrain 3D 384-well plate at a volume of 5μL per well. Activity was recorded after 1 week of incubation, to allow expression of the calcium indicator. For recording, we used ImageXpress Confocal Microscope (Molecular Devices) with a sampling rate of 25 frames per second for 500 seconds. Multiple Regions of Interest (ROI), representing different neurons on the neurospheroid, were selected for analysis on MetaXpress software (Molecular Devices). Recorded activity was plotted as Relative Fluorescence Units (RFU) vs Time (in seconds) using GraphPad Prism version 7.

### Screening assay

Three half-washes were performed on 3D neurosphere plates with phenol red-free BrainPhys Medium (StemCell Technologies, 05791) supplemented with 1x SM1, 20ng/mL of BDNF, 20ng/mL of GDNF, and 1x pen/strep. Next, Calcium 6 Dye (Molecular Devices, R8190) was added to a concentration of 1x and incubated for 2 hours at 37°C in 5% CO_2_ in preparation for the baseline reading. The baseline signal was read for 10 minutes on a FLIPR Tetra^®^ (Molecular Devices) with a capture rate of 2 Hz. Compounds were diluted in the above medium. After the baseline reading, compounds were added to the neurospheres and activity was measured after 30 minutes, 2 hours, and 4 hours for 10 minutes with a capture rate of 2 Hz.

### Compound library

The LOPAC^®1280^ library was purchased from Millipore-Sigma, and all compounds were solubilized in DMSO. In the primary screen, each compound was tested in duplicate (on separate plates) at 1 μM concentration. For concentration-response experiments, fresh powders were ordered and tested in ten-point half-log concentration intervals. For concentration response curves, each dilution was tested in triplicate.

### Data analysis

FLIPR Tetra^®^ data were analyzed using ScreenWorks PeakPro Software (Molecular Devices). Spatial uniformity correction was enabled, and peak detection was utilized with the following parameters: smooth width = 5, fit width = 10, and dynamic amplitude threshold = 20. Each well was normalized to its baseline reading before compound addition. Data were graphed using GraphPad Prism version 7. In the primary screen, compounds were counted as hits only if both replicates were greater or less than 2 times the standard deviation of the DMSO treated wells. Significant differences between vehicle and compound treated wells were determined by one-way ANOVA followed by Dunnett’s test. GraphPad Prism version 7 was used to perform all statistical analyses.

## Results

### 3D neurospheres display synchronized calcium oscillations

**Fig 1:**
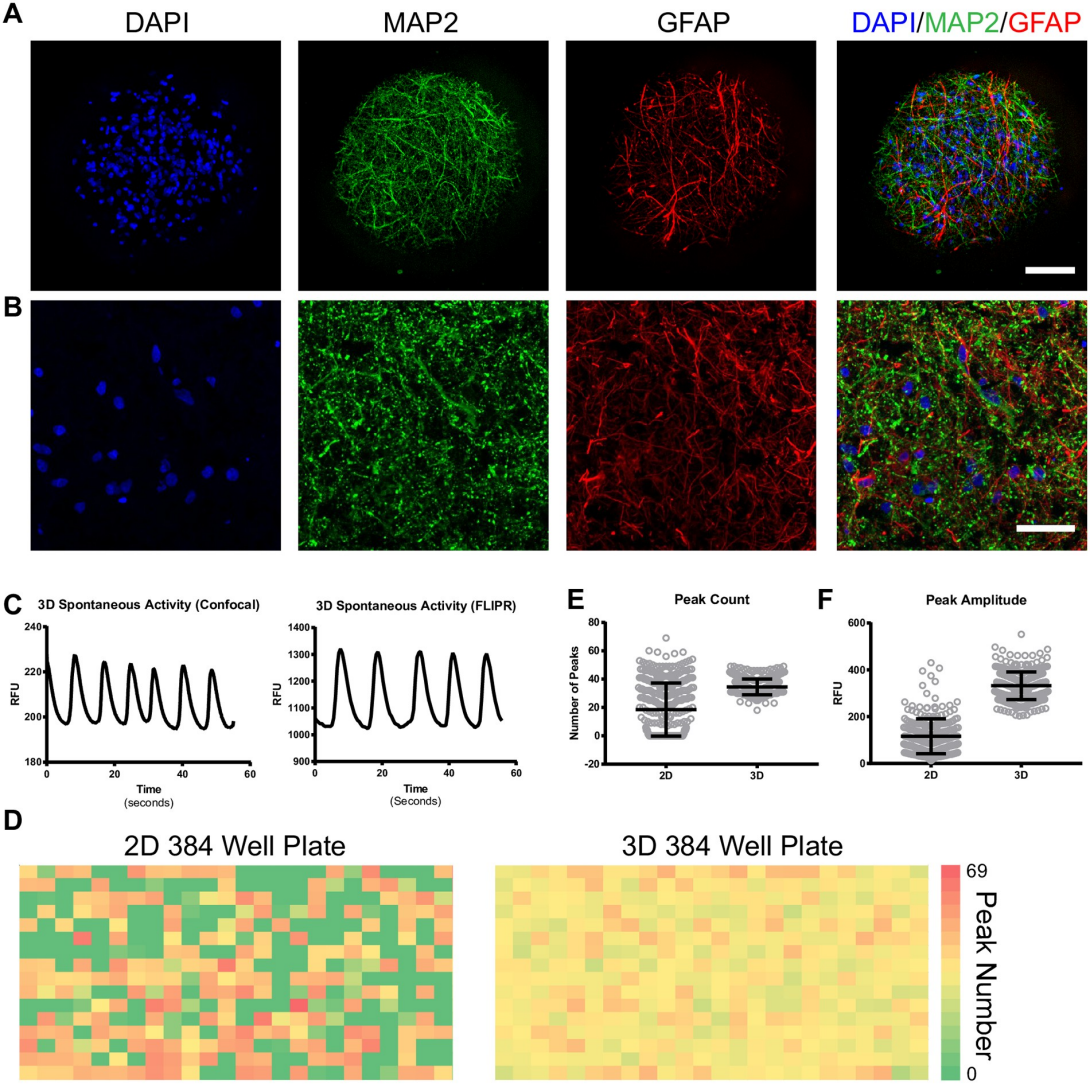
3D neurospheres contain neurons and astrocytes and generate highly synchronized network activity. A) Confocal image of a 3D neurosphere showing nuclei (DAPI-blue), neurons (MAP2-green), and astrocytes (GFAP-red) demonstrating the presence of a mixture of neurons and astrocytes on the spheroid surface. B) Cryosectioned 3D sphere showing nuclei (DAPI-blue), neurons (MAP2-green) and astrocytes (GFAP-red) illustrating the presence of these cells in the interior of the spheroid. Scale bars represent 50 μm. C) Quantification of calcium dye intensity over time in a 3D culture by confocal imaging and by FLIPR. RFU = relative fluorescence units. D) Heat map of peak frequency (peak count per 10min interval) in 384 well plate from 2D and 3D cultures. E) Mean peak count across wells of 2D and 3D plates. F) Mean peak amplitude across wells of 2D and 3D plates. Error bars represent standard deviation.

Two-dimensional (2D) cultures and 3D spheroids of human iPSC-derived cortical neurons and astrocytes were obtained from StemoniX, Inc. The presence of astrocytes and neurons were confirmed by positive staining with MAP2, a protein highly expressed in neurons, and GFAP, a protein expressed by astrocytes. We confirmed the distribution of neural cells on the surface (Fig 1A) and interior of the neurospheres (Fig 1B). As reported before for cultures of primary rodent neurons [12, 13], we found that these neurospheres display spontaneous activity that can be visualized with a calcium dye and that activity increased with culture age over time in culture (S1 Fig). Initial experiments utilized fluorescence microscopy for imaging calcium activity (Fig 1B), however the platform was quickly switched to a 384-well fluorometric imaging plate reader (FLIPR Tetra^®^) as this platform is more amenable to measuring compound-induced effects on the calcium waveform properties (such as oscillation frequency, amplitude, peak rise time, peak decay time) in a high-throughput fashion (Fig 1C).

One benefit of the neurospheres used in this study is their mixed composition of cortical neurons and astrocytes, making them more physiologically relevant than a pure neuronal culture. As functional activity could arise from either or both cell populations, we sought to confirm the neuronal contribution by limiting the calcium indicator presence to only neurons. To this end, we transduced neurospheres with a calcium indicator driven by the neuronal-specific synapsin promoter (S2 Fig). Spontaneous activity was detected by the synapsin-driven Ca^2+^ indicator, and the frequency pattern was similar to that of the ubuiquitous Ca^2+^ indicator used in the FLIPR recordings, thus confirming that neuronal activity contributes to at least a portion of the whole spheroid signal. The ability to isolate activity to a single cell population is important in that it could also be used in follow-up screens to elucidate cellular and molecular mechanisms of action triggered by unknown compounds. We next compared the spontaneous activity generated by cells grown in 2D cultures to that of the 3D cultures. Activity heat maps for peak count in 384-well plates of 2D and 3D cultures are shown in Fig 1C. In the 2D plate, 149 out of 384 wells (39%) did not show any detectable calcium oscillations (dark green squares) while the 3D plate, in comparison, showed detectable activity in 100% of the wells. Well to well consistency in oscillatory activity was also more reproducible in the 3D plate, with peak count ranging from 18 to 49 peaks over ten minutes (34.5 ± 5.5, mean ± s.d.) in the 3D plates versus 0 to 69 peaks over ten minutes (18.5 ± 18.7, mean ± s.d.) in the 2D plates (Fig 1D). Similarly, the consistency and magnitude of peak amplitude measured in relative fluorescence units (RFU) was greater in the 3D cultures (332.1 ± 59.0, mean ± s.d.) compared to 2D cultures (116.4 ± 74.7, mean ± s.d.; Fig 1E). Thus, the spontaneous activity generated by 3D cultures was more homogenous and robust from well-to-well than that of the 2D cultures, making the 3D platform more suitable for screening.

### 3D neurospheres respond to excitatory and inhibitory tool compounds

**Fig 2:**
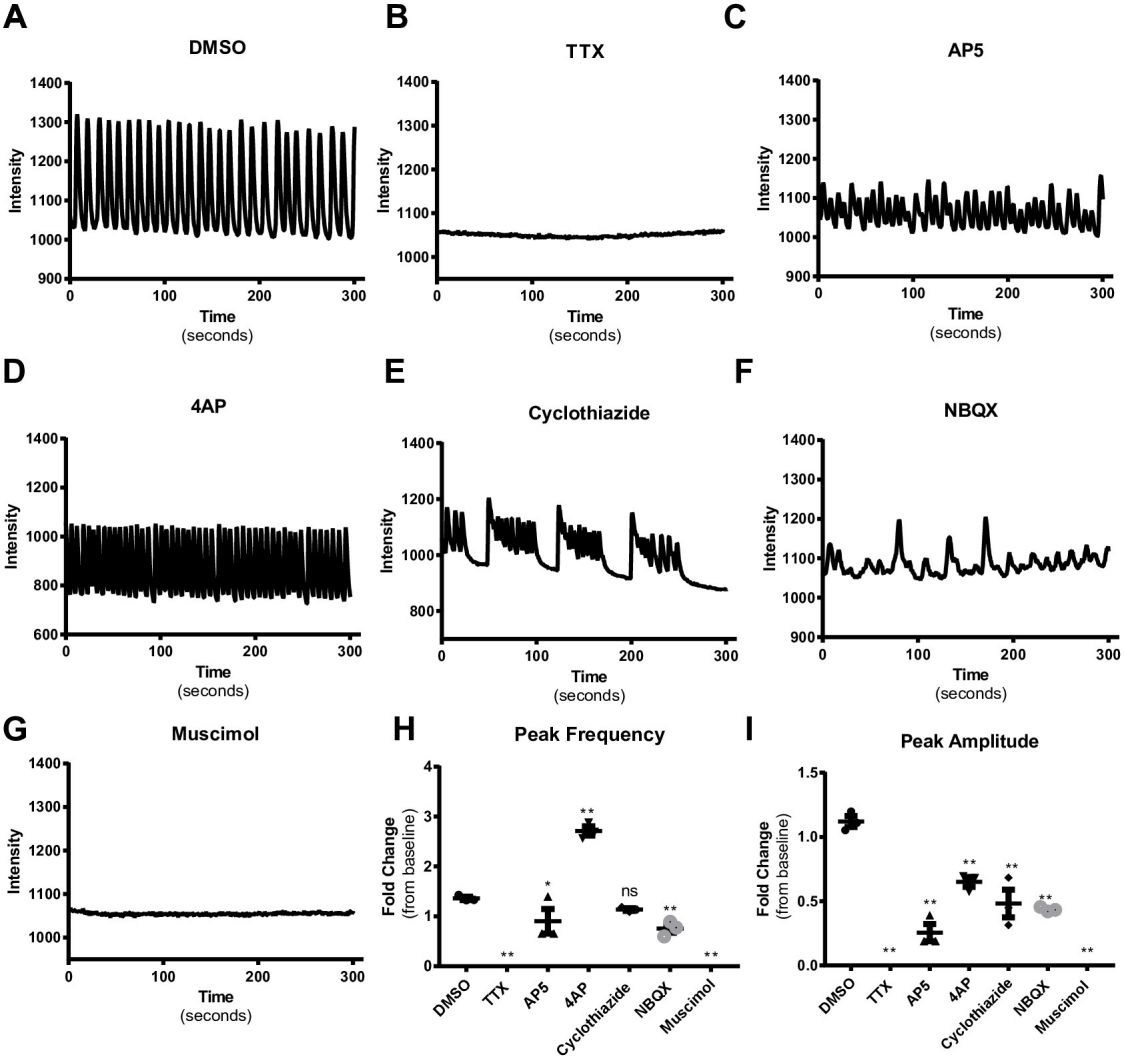
3D neurospheres respond to known excitatory and inhibitory reference compounds. Representative tracings of calcium dye intensity over time in single wells treated with DMSO or 1 μM of test compound as indicated in each panel (A-G). H) Quantification of peak frequency from compound treated wells. Each point represents an individual well with three wells per treatment. TTX, AP5 NBQX, and muscimol significantly decreased peak frequency compared with DMSO. 4AP significantly increases peak frequency compared with DMSO. Cyclothiazide and bicuculline did not significantly affect peak frequency compared to DMSO. Error bars represent SEM. I) Quantification of peak amplitude from compound treated wells. TTX, AP5, 4AP, cyclothiazide, NBQX, and muscimol all significantly decrease peak amplitude compared with DMSO. Bicuculline does not significantly affect peak amplitude compared to DMSO. Each point represents an individual well with three wells per treatment. Error bars represent SEM. * indicated P<0.05, ** indicates P<0.01, ns indicates P>0.05.

We next tested whether the spontaneous activity from 3D neurospheres could be modulated by reference compounds that activate or inhibit various ion channels and neurotransmitter receptors. Treating neurospheres with 1 μM tetrodotoxin (TTX), a potent voltage-gated sodium channel inhibitor, completely blocked spontaneous activity (Fig 2B, 2H and 2I). Treatment with 1 μM AP5, a selective NMDA receptor antagonist, decreased both the peak frequency and amplitude (Fig 2H and 2I) but did not completely block activity (Fig 2C). Treatment with 1 μM 4-aminopyridine (4AP), a K^+^ channel inhibitor, significantly increased peak frequency (Fig 2D and 2H) and decreased peak amplitude (Fig 2I). Treatment with 1 μM cyclothiazide, a positive allosteric modulator of AMPA receptors, induced seizure-like activity (Fig 2E) characterized by bursts of rapid and irregular activity with decreased peak amplitude and minimal changes in overall peak frequency (Fig 2H and 2I). Treatment with 1 μM NBQX, an AMPA receptor antagonist, significantly reduced both peak frequency and peak amplitude (Fig 2H and 2I) but did not completely block activity (Fig 2F). Finally, treatment with 1 μM muscimol, a GABA_A_ receptor agonist, completely blocked spontaneous activity (Fig 2G, 2H and 2I). Taken together, these findings demonstrate that excitatory activity is mediated by both NMDA and AMPA receptors, as antagonists of either class of receptors modulated but did not completely block spontaneous activity. Since the GABA_A_ receptor agonist muscimol blocked all neuronal activity, this suggests that GABA receptors are present and can mediate inhibitory activity.

### Screen with 1280 pharmacologically active compounds

To determine the potential utility of this platform for screening purposes, we performed a small screen with 1280 active compounds (LOPAC^®1280^) tested in duplicate at 1 μM concentration. Neurospheroid activity was measured via calcium fluctuations at 10-minute intervals before compound addition and at 30 minutes, 2 hours, and 4 hours following compound addition. We aimed to identify compounds that could increase or decrease activity and thus selected hits based on both replicates being greater than or less than two-times the standard deviation of the DMSO treated wells. We analyzed two parameters, peak frequency and peak amplitude, across vehicle controls and LOPAC^®1280^ compounds. Low variability for both parameters was observed in vehicle control (DMSO) wells within and across all 384-well plates for all timepoints (Table 1).

**Table 1:** Standard deviation of peak frequency and average peak amplitude at each time point in DMSO treated wells from each 384 well plate.

DMSO STDV (%)	Peak Frequency			Average Peak Amplitude		
	30 Mins	2 Hours	4 Hours	30 Mins	2 Hours	4 Hours
**Plate 1 –Rep 1**	15.5	13.8	13.7	25.0	19.0	18.2
**Plate 1 –Rep 2**	14.5	11.3	9.4	20.9	14.1	15.2
**Plate 2 –Rep 1**	15.3	10.6	11.0	24.0	16.6	16.2
**Plate 2 –Rep 2**	15.6	11.1	11.4	21.2	15.1	14.9
**Plate 3 –Rep 1**	13.5	15.6	15.4	21.5	27.7	14.6
**Plate 3 –Rep 2**	15.1	14.8	15.3	22.9	14.9	14.5
**Plate 4 –Rep 1**	13.7	14.1	13.9	25.0	17.4	16.2
**Plate 4 –Rep 2**	15.3	12.0	12.2	22.0	15.2	15.4

**Fig 3:**
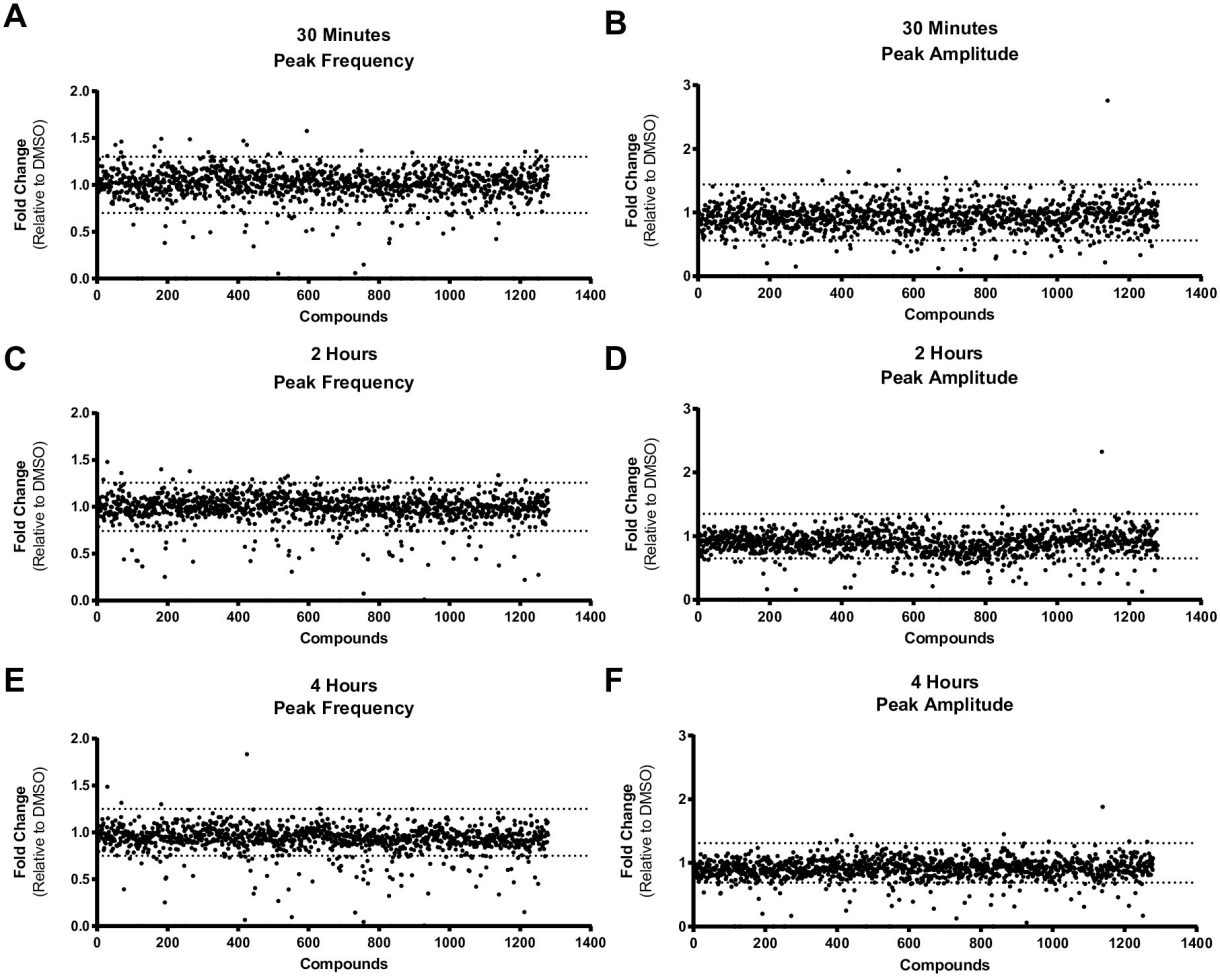
Screen with 1280 pharmacologically active compounds. Scatter plots of peak frequency at A) 30 minutes, C) 2 hours, and E) 4 hours after compound addition. Scatter plots of peak amplitude at B) 30 minutes, D) 2 hours, and F) 4 hours after compound addition. Each dot represents the average of duplicate wells relative to DMSO treated wells. Dashed lines indicate +/- two-times the standard deviation of the DMSO wells. Compounds that fell above or below dashed lines were classified as hits.

**Fig 4:**
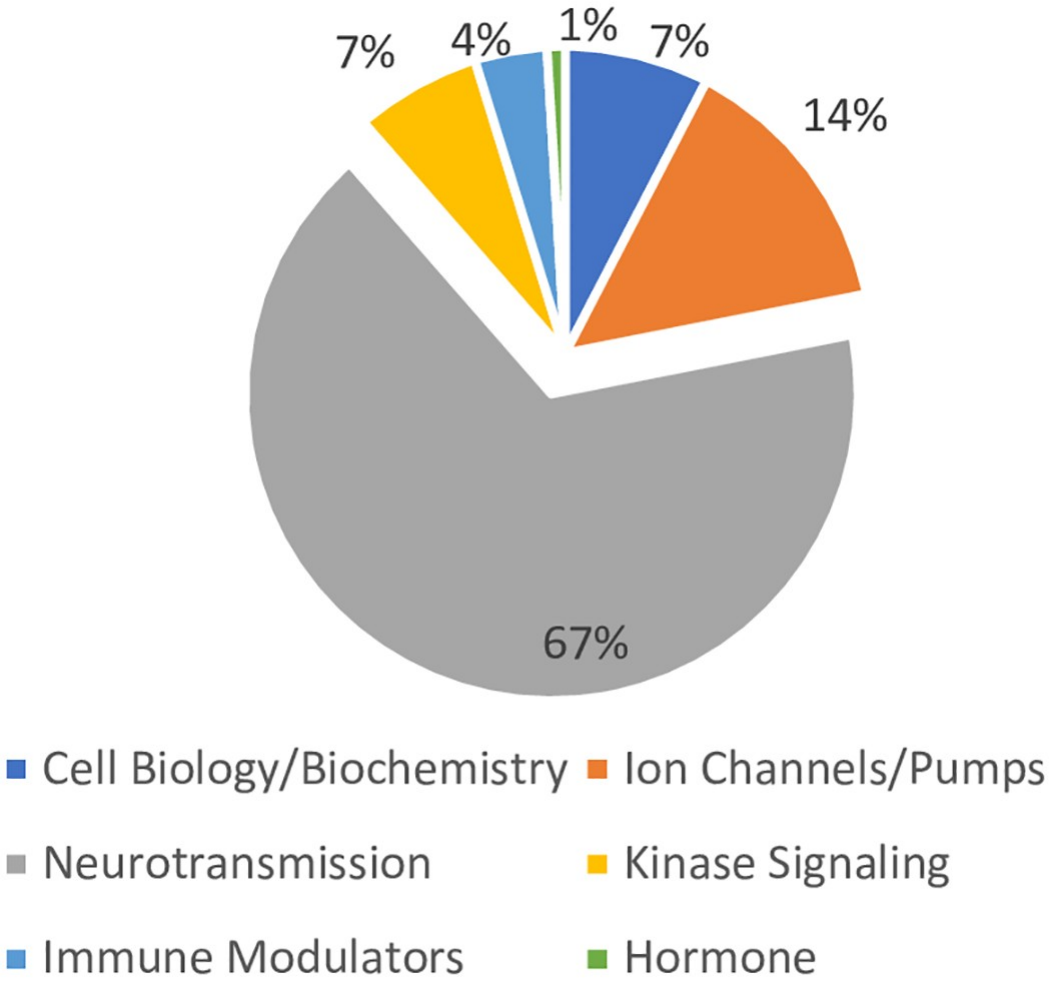
Distribution of hits from the LOPAC^®1280^ screen. Cellular pathways impacted by the hits are shown as a percent of the total 111 hits.

We defined a compound as hit when it changed neural activity (either peak frequency or amplitude) by twice the respective standard deviation of the negative control values; corresponding to 30% (peak frequency) and 40% (peak amplitude) changes. Scatter plots for each time point and peak frequency/amplitude are shown in Fig 3. In total, 111 compounds significantly affected peak frequency or amplitude on at least one timepoint with 17 of the 111 hits (15%) affecting both peak frequency and amplitude at all timepoints measured (Table 2) suggesting that many compounds presented a transient neural effect, while others have a longer lasting modulatory effect. In addition, 40 compounds (36%) only changed frequency or amplitude at 1 timepoint. Most of the hits, 100 out of 111 (90%), decreased peak frequency. Of the 11 compounds that increased peak frequency, only two (amitriptyline hydrochloride and forskolin) increased peak frequency at all three time-points (Table 2). Overall the hits spanned many different cellular processes (Fig 4). Most of the hits (58%) impacted neurotransmission-related pathways, while the rest affected diverse processes including (but not limited to) ion channels, cell biology/biochemistry, kinase signaling, as well as immune and hormone modulators (Fig 4). The diversity of hits, as well as the reproducibility of the vehicle controls, in this small-scale screen clearly validates the potential of this platform for identifying modulators of neuronal activity.

**Table 2:** Summary of all hits at 30 minutes, 2 hours, and 4 hours after compound addition for both peak frequency and amplitude.

Compound Name		Peak Count			Peak Amplitude		
	Compound Description	30 Min	2 Hour	4 Hour	30 Min	2 Hour	4 Hour
(-)-MK-801 hydrogen maleate	NMDA receptor antagonist	0.54					0.49
(-)-Naproxen sodium	COX-1 and COX-2 inhibitor						0.68
(-)-Physostigmine	Cholinesterase inhibitor			0.74			
(-)-trans-(1S,2S)-U-50488 hydrochloride	Opioid receptor agonist		0.67	0.58			
(+)-Butaclamol hydrochloride	Dopamine receptor antagonist					0.55	0.59
(+)-MK-801 hydrogen maleate	NMDA receptor antagonist	0.38	0.43	0.32	0.27	0.26	0.34
(+)-Quisqualic acid	Activates glutamate receptors	0	0.27	0.45	0	0.12	0.17
(±)-2-Amino-4-phosphonobutyric acid	NMDA receptor antagonist	1.42					
(±)-8-Hydroxy-DPAT hydrobromide	5-HT1A serotonin receptor agonist	0			0		
(±)-alpha-Lipoic Acid	TrkA inhibitor		0.51				
(±)-AMT hydrochloride	NOSs inhibitor	1.46				0.57	
(±)-Butaclamol hydrochloride	Dopamine receptor antagonist					0.53	
(±)-Isoproterenol hydrochloride	Sympathomimetic amine acting on β adrenoceptors					0.44	0.54
(±)-Propranolol hydrochloride	β Adrenoceptor antagonist	0			0	0.29	0.31
2-Chloroadenosine	Adenosine receptor agonist	0	0	0	0	0	0
2-Chloroadenosine triphosphate tetrasodium	P2Y receptor agonist	0.53		0.26			
3-alpha,21-Dihydroxy-5-alpha-pregnan-20-one	Modulator of GABA-A	0.59			0.36	0.35	0.56
4-[5-[[4-Oxo-3-(phenylmethyl)-2-thioxo-5-thiazolidinylidene]methyl]-2-furanyl]-benzoic acid	CD11b/CD18 agonist					0.64	
5-(N,N-Dimethyl)amiloride hydrochloride	Blocks Na^+^/H^+^ antiport				0.5		
5,5-Diphenylhydantoin	Anticonvulsant	1.57					
5alpha-Pregnan-3alpha-ol-20-one	Modulator of GABA	0.53	0.44	0.62	0.39	0.48	0.57
5-Carboxamidotryptamine maleate	5-HT7 Serotonin receptor agonist			0.74			
5-hydroxydecanoic acid sodium	Blocks K^+^ channel	0			0		
AC-93253 iodide	RARalpha agonist		0.31	0.09		0.47	0.32
Aconitine	Activates tetrodotoxin-sensitive Na^+^ channels. Blocks norepinephrine reuptake	0	0	0	0	0	0
Alpha-Lobeline hydrochloride	Nicotinic acetylcholine receptor agonist				0.51	0.47	
Amitriptyline hydrochloride	Tricyclic antidepressant	1.49	1.4	1.3	.47	.41	0.43
Aprindine hydrochloride	H1 histamine receptor antagonist					0.57	
Arecaidine propargyl ester hydrobromide	Muscarinic acetylcholine receptor agonist		0.64	0.644			
Azelaic acid	Inhibits mitochondrial oxidoreductases and DNA synthesis	1.41					
Benoxathian hydrochloride	α1 adrenoceptor antagonist		0.43	0.39			0.52
BW 723C86	5-HT2B serotonin receptor agonist					0.55	
Calcimycin	Ca^2+^ ionophore	0.44	0.41	0.53	0.15	0.15	0.17
Carbamazepine	Analgesic; anticonvulsant	0.56					
cDPCP	Na+ channel blocker					0.46	
CGS-15943	A1 adenosine receptor antagonist						0.58
Clonidine hydrochloride	α2 Adrenoceptor agonist			0.66			
CNS-1102	Noncompetitive NMDA receptor antagonist	0.38	0.25	0.25	0.2	0.16	0.2
Cyclosporin A	Calcineurin phosphatase inhibitor		1.47	1.48			0.53
Dequalinium chloride hydrate	Blocks apamin-sensitive K^+^ channels	0.5			0.39		
Dihydroouabain	Sodium-potassium inhibitor	0	0	0	0	0	0
Dihydroouabain	Na^+^-K^+^ pump inhibitor				0.51		
Diphenyleneiodonium chloride	Endothelial NOSs inhibitor	0.49		0.06	1.63		0.56
DNQX	Kainate/quisqualate glutamate receptor antagonist				0.39		
Donitriptan monohydrochloride	5-HT1B/1D agonist			0.77			
Doxepin hydrochloride	Antidepressant					0.55	
Fluphenazine dihydrochloride	Dopamine receptor antagonist						0.58
Fluvoxamine maleate	Serotonin reuptake inhibitor					0.58	
Forskolin	Activates adenylate cyclase	1.42	1.36	1.83	0.48	0.19	0.25
FPL 64176	L-type Ca^2+^ channel activator	0.34		0.34			
Ganaxolone	Allosteric modulator of GABA-A receptors	0	0.56	0.47	0	0.39	0.39
GBR-12909 dihydrochloride	Dopamine reuptake inhibitor					0.6	
GR 79236X	Adenosine receptor agonist	0	0	0	0	0	0
GR-89696 fumarate	Opioid receptor agonist		0.64	0.67			
Harmane	I1 imidazoline agonist						1.35
HI-TOPK-032	TOPK inhibitor					0.6	0.51
Hydrochlorothiazide	Carbonic anhydrase inhibitor	0			0		
ICI 204,448 hydrochloride	Opioid receptor agonist			0.64			
Ipratropium bromide	Muscarinic acetylcholine receptor antagonist			0.71			
Ivermectin	Modulator of α7 and GABA	0	0	0	0	0	0
JS-K	Nitric oxide donor				1.66		
Kainic acid	Excitatory amino acid receptor agonist				0.38		0.63
Lamotrigine	Anticonvulsant	0			0		
Leflunomide	Immunosuppressive				0.41		
Loperamide hydrochloride	Ca^2+^ channel antagonist	0.15	0.07	0.04			
Lubeluzole dihydrochloride	NMDA receptor antagonist					0.38	0.42
Lubiprostone	Activates ClC-2 and CFTR chloride channels	0.59		0.53			
Lumefantrine	Antimalarial drug						0.6
Methiothepin mesylate	5-HT1 Serotonin receptor antagonist					0.51	
Methoctramine tetrahydrochloride	M2 muscarinic acetylcholine receptor antagonist					0.54	
Mibefradil dihydrochloride	T-type Ca^2+^ blocker			0.57			
Moxonidine hydrochloride	α2A adrenoreceptor agonist	0.56	0.47	0.5			
N6-Cyclohexyladenosine	Adenosine receptor agonist	0	0	0	0	0	0
N6-Cyclopentyladenosine	Adenosine receptor agonist	0	0	0	0	0	0
NBQX disodium	AMPA/kainate glutamate receptor antagonist	0.43	0.49	0.52	0.31	0.34	0.49
Niclosamide	Protonophoric anthelmintic	0.06	0	0.14	0.73	0	0.12
N-Methyl-1-deoxynojirimycin	Inhibits glucosidase	0			0		
NS8593 hydrochloride	Blocks Ca^2+^ activated K^+^ channels					0.66	
Ouabain	Blocks Na^+^-K^+^ ATPases	0	0	0	0	0	0
Oxotremorine methiodide	Muscarinic acetylcholine receptor agonist	0.56	0.58	0.54			1.45
Oxotremorine sesquifumarate salt	Muscarinic acetylcholine receptor agonist			0.54			
Palmitoyl-DL-Carnitine chloride	Modulates PKC	0			0		
p-Aminoclonidine hydrochloride	α2 adrenoceptor agonist	0.56	0.55	0.5			
PD-407824	Wee1/Chk1 inhibitor					0.56	
Phorbol 12-myristate 13-acetate	Activates protein kinase C; strong NO promoter	0.59	0.37	0.33	2.76	2.32	1.88
Pimozide	Ca^2+^ and D2 dopamine receptor antagonist			0.52			
p-Iodoclonidine hydrochloride	α2 adrenoceptor agonist			0.59			
Propionylpromazine hydrochloride	Inhibitor of hedgehog acyltransferase			0.51		0.42	
Psora-4	Potassium channel inhibitor	1.58	1.38				
Quinidine sulfate	D2 dopamine receptor antagonist					0.2	
R(-)-Me5	Na channel antagonist					0.45	0.67
R(-)-N6-(2-Phenylisopropyl)adenosine	A1 Adenosine receptor agonist	0	.01		0		0.06
Reserpine	Inhibits vesicular catecholine and serotonin uptake		0.61	0.51			
Ro 25–6981 hydrochloride	NMDA receptor antagonist	0.48	0.61		0.31	0.46	
RS504393	CCR2 chemokine receptor antagonist			0.346			
RU-SKI 43 maleate	Platinum-based antineoplastic agent			0.51			
S-(-)-Eticlopride hydrochloride	D2 receptor antagonist						0.65
SB 216763	Inhibitor of GSK-3	0			0		
SDZ 220–581 hydrochloride	NMDA receptor antagonist	0.47			0.87	0.21	0.28
Terfenadine	H1 histamine receptor antagonist			0.6			
Tetracaine hydrochloride	Local anesthetic	0.42			0.21	0.26	
TIC10 angular	Induces TRAIL					0.59	
Tizanidine hydrochloride	α2-adrenoceptor agonist		0.68	0.6			
Trifluperidol hydrochloride	Dopamine receptor antagonist	0.67		0.62	0.45	0.57	
Tyrphostin AG 879	Class Ib antiarrhythmic and hERG channel blocker		0.7				
U-62066	Opioid receptor agonist		0.59	0.59			
UK 14,304	α2 Adrenoceptor agonist	0	0.22	0.14	0	0.25	0.32
Vinpocetine	PDE1 inhibitor				0.33	0.45	0.52
WB-4101 hydrochloride	α1A Adrenoceptor antagonist	0.66	0.52		0.37	0.51	
Wiskostatin	Inhibitor of N-WASP	1.35					
YM 976	PDE4 inhibitor					0.47	0.73

Values are fold-change from DMSO treated wells and indicate that frequency and/or amplitude was greater or less than two-times the standard deviation from DMSO. No value corresponds to no change from DMSO treated wells.

### Hit confirmation and concentration response with select compounds

**Fig 5:**
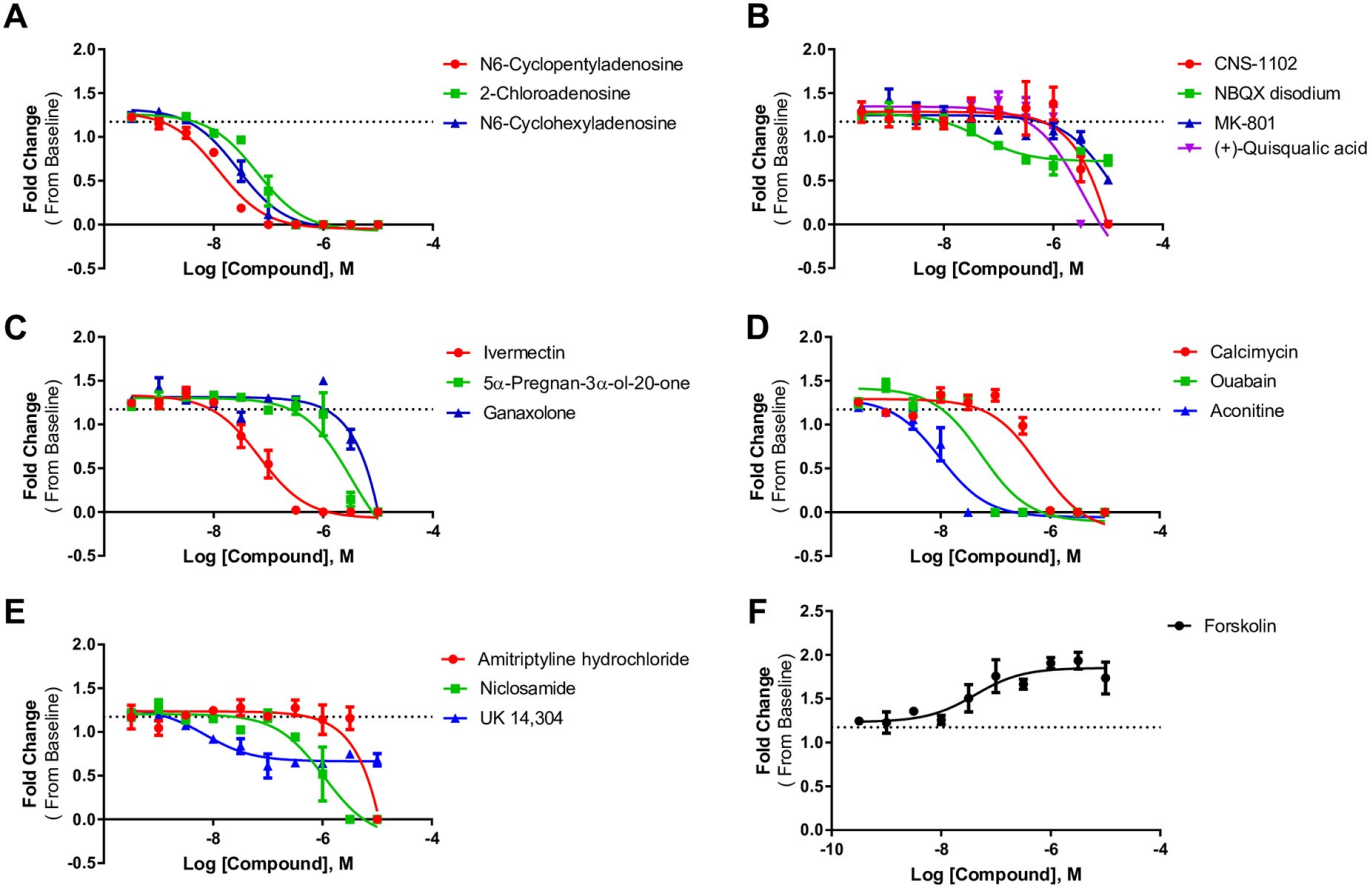
Concentration response curves of peak frequency. A) Adenosine receptor agonists, B) glutamate receptor compounds, C) GABA receptor compounds, D) blockers of various channels, E) other compounds, and F) concentration response of forskolin. Each point represents n = 3 wells. The dashed line on each graph represents the average of the DMSO-treated wells.

**Fig 6:**
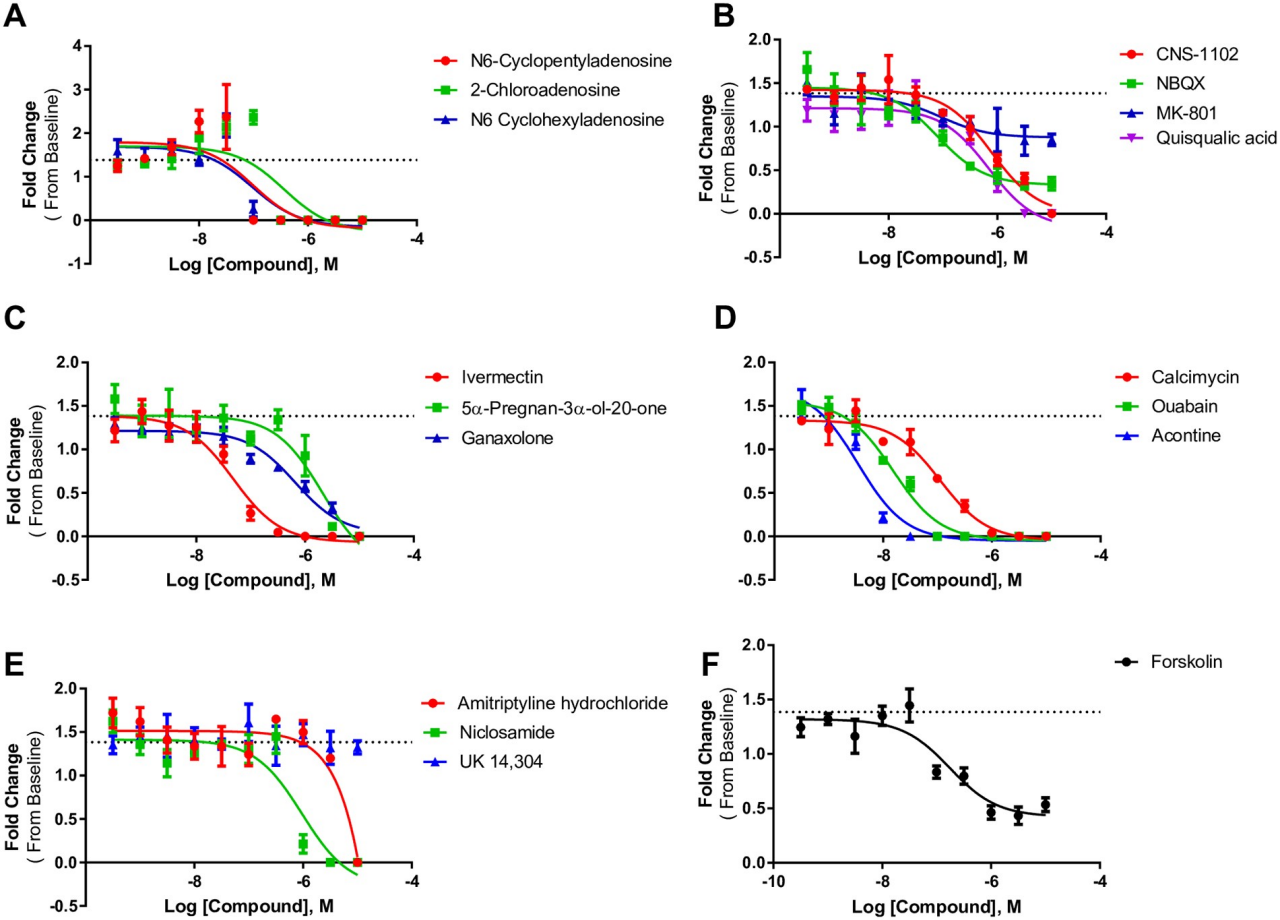
Concentration response curves of peak amplitude. A) Adenosine receptor agonists, B) glutamate receptor compounds, C) GABA receptor compounds, D) blockers of various channels, E) other compounds, and F) concentration response of forskolin. Each point represents n = 3 wells. The dashed line on each graph represents the average of the DMSO-treated wells.

We selected 17 compounds for further confirmation based on their ability to alter peak frequency and amplitude at the majority of tested timepoints. Of the 17 compounds tested in concentration response, three are adenosine receptor agonists, four are antagonists of NMDA or AMPA receptors, and three are positive allosteric modulators of GABA receptors. The remaining seven compounds act on various ion channels and other cellular processes (Table 3). Concentration response curves for peak frequency and amplitude for each compound at the 30-minute timepoint are displayed in Figs 5 and 6. EC50s for each compound at each timepoint are listed in Table 3. Of the 17 compounds tested, 16 were confirmed active in concentration response assays. Notably, the adenosine receptor agonists had effects in the low nanomolar range. The only compound that was not confirmed was amitriptyline hydrochloride, which is a tricyclic antidepressant. This compound increased peak frequency ~1.5 fold and decreased peak amplitude ~0.5 fold in the primary screen at 1 μM (Table 2), but when tested in a concentration-response, it did not increase frequency and blocked all spontaneous activity at the highest concentrations (Fig 5E). Forskolin was the only compound confirmed to increase peak frequency with an EC_50_ of 45 nM at 30 minutes (Table 3). Forskolin also decreases peak amplitude at 30 minutes with an EC_50_ of 45 nM at 30 minutes (Table 3).

**Table 3:** EC_50_ values for each of the 17 compounds tested in the concentration response experiment at 30 minutes, 2 hours, and 4 hours after compound addition.

Compound Name	Compound Description	Peak Frequency EC_50_			Peak Amplitude EC_50_		
		30 Min	2 Hours	4 Hours	30 Min	2 Hours	4 Hours
(+)-MK801 hydrogen maleate	NMDA receptor antagonist	1.18μM	70.7nM	53nM	90.8nM	18.9nM	>10μM
(+)-Quisqualic acid	AMPA and kainate agonist	3.45μM	4.66μM	4.82μM	693nM	800nM	1.21μM
2-Chloroadenosine	Adenosine receptor agonist	62.1nM	320nM	264nM	363nM	2.51μM	1.4μM
Aconitine	Activates tetrodotoxin-sensitive Na^+^ channels	9.88nM	12.3nM	23.5nM	3.48nM	9.23nM	21.1nM
Allopregnan-3alpha-ol-20-one	Modulator of GABA	3.25μM	9.4μM	>10μM	2.13μM	2.23μM	3.61μM
Amitriptyline hydrochloride	Tricyclic antidepressant	>10μM	>10μM	>10μM	1.76μM	6.7μM	4.0μM
Calcimycin	Ca^2+^ ionophore	625nM	680nM	694nM	112nM	83.3nM	72.3nM
CNS-1102	Noncompetitive NMDA receptor antagonist	>10μM	3μM	2.93μM	732nM	632nM	476nM
Forskolin	Activates adenylate cyclase	41.3nM	277nM	407nM	43.6nM	145nM	183nM
Ganaxolone	Allosteric modulator of GABA receptors	>10μM	>10μM	>10μM	2.52μM	1.7μM	2.7μM
Ivermectin	Modulator of α7 and GABA	68.1nM	22.3nM	15nM	48.0nM	20.2nM	15nM
N6-Cyclohexyladenosine	Adenosine receptor agonist	27.7nM	77.2nM	696nM	94.2nM	379nM	324nM
N6-Cyclopentyladenosine	Adenosine receptor agonist	12.3nM	47.3nM	38.7nM	98.4nM	302nM	119nM
NBQX Disodium	AMPA/kainite glutamate receptor antagonist	47.9nM	116nM	119nM	79.1nM	533nM	106nM
Niclosamide	Protonophoric anthelmintic	1.06μM	3.0μM	2.51μM	954nM	943nM	923nM
Ouabain	Blocks Na^+^, K^+^ ATPases	55.7nM	32.8nM	18.7nM	15.4nM	9.1nM	12.0nM
UK 14,304	α2 Adrenoceptor agonist	7.11nM	29.8nM	11.1nM	>10μM	>10μM	>10μM

## Discussion

Development of a high-throughput, functional readout for neuronal network activity has been a challenge over the years, and a few methods of assessing neuronal function in a high-throughput format have recently been described. The MANTRA™ system developed by the Galenea Corporation uses a pH-sensitive synaptic reporter to monitor synaptic vesicle cycling as a readout for neuronal activity [17]. Virdee et al. reported on a similar technology using a calcium sensitive dye to measure responses in networks of cultured rat neurons *in vitro* [18]. Both systems measure responses evoked by electrical field stimulation in 2 dimensional primary rodent neurons. In this study we extend these investigations to assess human-based neural activity using iPSC-derived 3D neurospheroids with the goal of developing a higher-throughput functional screening platform. Similar to previous work we show that functional activity can be monitored via a calcium sensitive dye [18]. Further, we provide several lines of evidence supporting a neuronal contribution to the whole spheroid Ca^2+^ signal including; 1) using a synapsin promotor-driven Ca^2+^ fluorophore to demonstrate temporal correlation between neuronal specific and whole spheroid Ca^2+^ signals, 2) demonstrating neuronal connectivity by showing synchronization of the neuronal-specific Ca^2+^ signals across discrete areas of the neurospheroid, and lastly 3) modulating the whole neurospheroid Ca^2+^ signal with neuronal specific ion channel and neurotransmitter agonists and antagonists. While not ruling out other sources, e.g. astrocytes, these data and that of others [19] provides strong evidence for a neuronal contribution to the whole neurospheroid Ca^2+^ signal.

Synchronous bursting of neurons in culture is believed to be a result of spontaneous miniature synaptic conductances in combination with random depolarizations that exert an intrinsic timing of bursting in the network. Though the exact mechanism of spontaneous network bursting is not known, changes in the frequency and amplitude of calcium transients associated with these network bursts likely represent many aspects of synaptic transmission and the activity of many neurotransmitter systems and are thus suitable surrogate phenotypic marker for neural activity. This may be an advantage in a compound or genetic screening since hits have the potential to affect a broad number of pathways and mechanisms of neurotransmission and phenotypic-based screening is gaining traction as a successful approach in drug-discovery [16]. The potential utility of simultaneously interrogating multiple pathways with a phenotypic approach was demonstrated here with the results showing that the diverse compound collection of the LOPAC^®1280^ library impacted diverse cellular processes of the human neural spheroids.

Traditionally, incorporating phenotypic and/or native functional readouts from 2-dimensional cultures has been difficult in screening paradigms has been difficult. Similarly, we observed greater well-to-well variation across the 2D plates used in these experiments. One potential reason for the greater well-to-well variation in the 2D cultures is that the amplitude of the oscillations could be below the detection limit of our instrument. However, this still highlights the utility of the consistent activity of the 3D cultures that was detected in every well. The observed very low well-to-well variability on 3D neurospheres illustrates how they are able to bring native function into high-throughput screening applications. By analyzing the frequency and amplitude of calcium oscillations in these 3D cultures of human neurons and astrocytes, we were able to screen for modulators of network activity. Features of the calcium oscillations such as frequency, amplitude, and synchronization can be used to describe network activity and quantified to rank the effects of various compounds on the system. We demonstrated that spontaneous activity can be modulated with known excitatory and inhibitory compounds. Subsequently, a library of 1280 compounds with known activity was screened at 1 μM, and 111 (8.6%) compounds were identified that modulated neuronal network activity through a wide variety of biological mechanisms. Of these, 17 compounds were tested in concentration response experiments, and 16 of the 17 compounds were confirmed as active, including three classes of compounds that affected peak frequency and amplitude at all time points: adenosine receptor agonists, GABA receptor modulators, and glutamate receptor modulators. Additionally, seven other compounds acting through various mechanisms inhibited spontaneous activity. It is possible that some compounds identified in our screen are toxic and thus decrease activity in a non-specific manner. While we did not perform a viability assay due to the acute nature of the treatment, this could be done either simultaneously or as a follow-up exercise to address cell viability with novel hits in subsequent screens similar to previous work using longer incubation periods [19]. There were two compounds in the primary screen, forskolin and amitriptyline hydrochloride, that increased peak frequency at all timepoints. Forskolin activates adenylate cyclase and thus likely increases spontaneous activity in a non-specific manner. Amitriptyline hydrochloride is a tricyclic antidepressant and was the only compound that was not confirmed in the secondary concentration-response experiment. In the primary screen, amitriptyline hydrochloride increased peak frequency, but when fresh powder was obtained and the experiment repeated for concentration response, this compound failed to repeat and abolished spontaneous activity at the highest concentration.

One advantage of screening neurospheres system used on this study is the balanced composition of neurons and astrocytes, better mimicking the *in vivo* condition. The 3D system is more physiologically relevant as the two cell types exist in a more natural state. This may be a benefit in screening for novel compound modulators of network activity. While the compounds we tested here are well characterized and have known effects on neurons and neurotransmitter systems, any unknown compounds discovered that impact neurosphere activity could be specific to astrocytes or neurons. We presented one method for uncovering cell-specific effects through the use of a neuronal specific Ca^2+^ sensor. Moreover, the use of calcium reporters driven by targeted promoters could help to visualize the activity modulation on different cell types when under the influence of compounds.

Modeling disease using human iPSCs has revealed consistent synaptic deficits in several disorders including schizophrenia and autism [1, 4, 20]. The assay described herein may have the potential for future disease-modeling studies of psychiatric disorders and phenotypic drug screening. New therapies for neurological disorders are a vital area of unmet need partially due to the unknown complexity of the central nervous system. This 3D neurosphere platform may provide a phenotypic approach to interrogating neurological diseases and ultimately lead to therapies. The microBrain 3D neurospheres have also been used to profile neurotoxic compounds, which highlights the broad applicability of this platform [19].

In summary, we have described the utility of 3D human neural cultures in drug screening for modulators of neural network activity. We generated a data set that can serve as a reference for compounds that alter spontaneous network activity. Moreover, we confirmed that compounds that increase or decrease network bursting can be identified in a reproducible fashion using this assay, making it ideal for screening campaigns. This platform is robust and can be used for future drug screening and disease modeling efforts.

## Supporting information

S1 Fig Neurospheroid activity increases over time in culture. A) Number of Ca2+ peaks recorded from individual wells over a 10 minute window in 6, 8, 10 and 12week cultures. B) Mean, Standard Deviation (STD) and Coefficient of Variation (CoV) for peak count from 6, 8, 10 and 12-week cultures. C) Representative 10 minute (600 second) recordings from single spheres at 6, 8, 10 and 12-week cultures. RFU = relative fluorescence units.
(PDF)

S2 Fig Spontaneous Ca2+ activity on neurons from neurospheres. After transduction with a synapsin promoter driven calcium indicator, three Regions of Interest (ROI) were selected and spontaneous Ca2+ activity was recorded. ROIs tracings were aligned according to timestamp, showing synchronized Ca2+ activity in neurons spatially separated in the spheroid. Graphs show Relative Fluorescence Units (RFU) vs Time (in seconds). Spontaneous activity recorded for 500 sec using ImageXpress Confocal Microscope. Recordings were performed in 6-week neurospheres.
(PDF)
