# Supplementary material for: Screening for modulators of neural network activity in 3D human iPSC-derived cortical spheroids
Source: PLoS One. 2020 Oct 22;15(10):e0240991. doi: 10.1371/journal.pone.0240991 (PMC7581002; doi:10.1371/journal.pone.0240991)
Supplement: S1 Fig — A) Number of Ca2+ peaks recorded from individual wells over a 10 minute window in 6, 8, 10 and 12week cultures. B) Mean, Standard Deviation (STD) and Coefficient of Variation (CoV) for peak count from 6, 8, 10 and 12-week cultures. C) Representative 10 minute (600 second) recordings from single spheres at 6, 8, 10 and 12-week cultures. RFU = relative fluorescence units. (PDF) [file pone.0240991.s001.pdf]

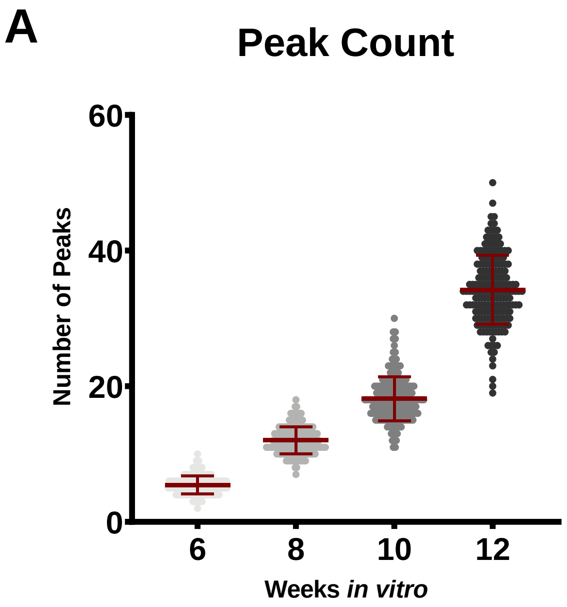


B

| Week | 6 | 8 | 10 | 12 |
|---|---|---|---|---|
| Mean | 5.448 | 12.03 | 18.15 | 34.21 |
| STD | 1.357 | 1.997 | 3.223 | 5.099 |
| CoV | 24.90% | 16.60% | 17.76% | 14.90% |

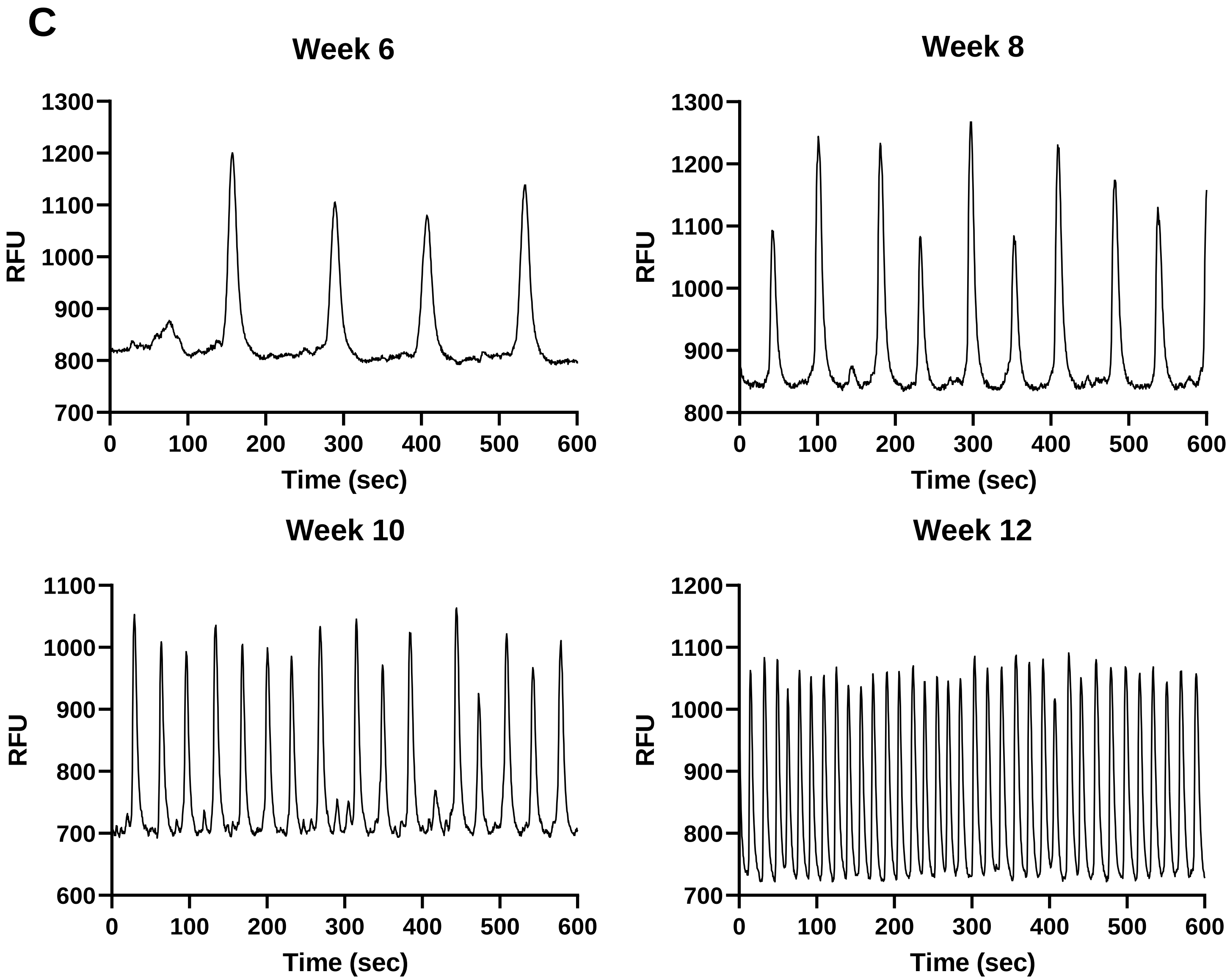


**S1 Fig.** Neurospheroid activity increases over time in culture. A) Number of $Ca^{2+}$ peaks recorded from individual wells over a 10 minute window in 6, 8, 10 and 12-week cultures. B) Mean, Standard Deviation (STD) and Coefficient of Variation (CoV) for peak count from 6, 8, 10 and 12-week cultures. C) Representative 10 minute (600 second) recordings from single spheres at 6, 8, 10 and 12-week cultures. RFU = relative fluorescence units.
