# Supplementary material for: Screening for modulators of neural network activity in 3D human iPSC-derived cortical spheroids
Source: PLoS One. 2020 Oct 22;15(10):e0240991. doi: 10.1371/journal.pone.0240991 (PMC7581002; doi:10.1371/journal.pone.0240991)
Supplement: S2 Fig — After transduction with a synapsin promoter driven calcium indicator, three Regions of Interest (ROI) were selected and spontaneous Ca2+ activity was recorded. ROIs tracings were aligned according to timestamp, showing synchronized Ca2+ activity in neurons spatially separated in the spheroid. Graphs show Relative Fluorescence Units (RFU) vs Time (in seconds). Spontaneous activity recorded for 500 sec using ImageXpress Confocal Microscope. Recordings were performed in 6-week neurospheres. (PDF) [file pone.0240991.s002.pdf]

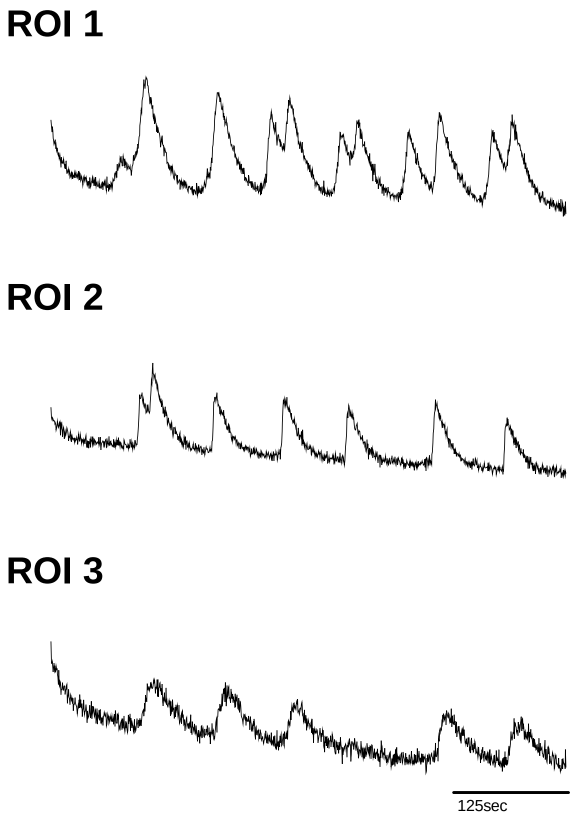

S2 Fig. Spontaneous Ca^{2+} activity on neurons from neurospheres. After transduction with a synapsin promoter driven calcium indicator, three Regions of Interest (ROI) were selected and spontaneous Ca^{2+} activity was recorded. ROIs tracings were aligned according to timestamp, showing synchronized Ca^{2+} activity in neurons spatially separated in the spheroid. Graphs show Relative Fluorescence Units (RFU) vs Time (in seconds). Spontaneous activity recorded for 500 sec using ImageXpress Confocal Microscope. Recordings were performed in 6-week neurospheres.
